# The mutant *Moonwalker* TRPC3 channel links calcium signaling to lipid metabolism in the developing cerebellum

**DOI:** 10.1093/hmg/ddv150

**Published:** 2015-04-23

**Authors:** Anna Dulneva, Sheena Lee, Peter L. Oliver, Katalin Di Gleria, Benedikt M. Kessler, Kay E. Davies, Esther B. E. Becker

**Affiliations:** 1 Medical Research Council Functional Genomics Unit,; 2 Department of Physiology, Anatomy and Genetics, University of Oxford, Oxford OX1 3PT, UK and; 3 TDI Mass Spectrometry Laboratory, Target Discovery Institute, University of Oxford, Oxford OX3 7FZ, UK

## Abstract

The *Moonwalker* (*Mwk*) mouse is a model of dominantly inherited cerebellar ataxia caused by a gain-of-function mutation in the transient receptor potential (TRP) channel TRPC3. Here, we report impairments in dendritic growth and synapse formation early on during Purkinje cell development in the *Mwk* cerebellum that are accompanied by alterations in calcium signaling. To elucidate the molecular effector pathways that regulate Purkinje cell dendritic arborization downstream of mutant TRPC3, we employed transcriptomic analysis of developing Purkinje cells isolated by laser-capture microdissection. We identified significant gene and protein expression changes in molecules involved in lipid metabolism. Consistently, lipid homeostasis in the *Mwk* cerebellum was found to be disturbed, and treatment of organotypic cerebellar slices with ceramide significantly improved dendritic outgrowth of *Mwk* Purkinje cells. These findings provide the first mechanistic insights into the TRPC3-dependent mechanisms, by which activated calcium signaling is coupled to lipid metabolism and the regulation of Purkinje cell development in the *Mwk* cerebellum.

## Introduction

Cerebellar ataxia defines a heterogeneous group of neurological disorders that affect the cerebellum and its afferent and efferent connections and manifest primarily as progressive gait and limb incoordination. The inherited forms of cerebellar ataxia encompass a growing list of genetically diverse disorders with more than 35 genetic loci known to cause the autosomal dominant ataxias alone, which are also referred to as spinocerebellar ataxias (SCAs) (1,2). Despite this genetic heterogeneity, common pathological mechanisms that may be shared among the different forms of cerebellar ataxia are beginning to emerge (2,3). Notably, disruption of intracellular calcium homeostasis and signaling in Purkinje cells has been proposed as a key mechanism in the pathogenesis of SCAs (1,4,5).

The transient receptor potential (TRP) channel TRPC3 is a non-selective cation channel that is highly expressed in the Purkinje cells of the cerebellum (6,7). TRPC3 is required for metabotropic glutamate receptor subtype 1 (mGluR1)-dependent synaptic transmission in Purkinje cells (6). Both genetic loss of *Trpc3* and the dominant *Moonwalker* (*Mwk*) gain-of-function point mutation in *Trpc3* result in cerebellar ataxia in the mouse (6,8), highlighting the importance of Purkinje cell calcium homeostasis for proper cerebellar function. Recently, we have identified the first functionally pathogenic variant (R672H) in the human *TRPC3* gene in a patient with adult-onset cerebellar ataxia (9). The human R672H mutation likely acts through a toxic gain-of-function mechanism similar to the *Mwk* mutation. Moreover, TRPC3 signaling has been linked to several other genetic forms of cerebellar ataxia in human and mouse including SCA1, SCA14, SCA15 and mutations in the GluD2 receptor (1) (reviewed in (7)). Thus, the disruption of the TRPC3 signaling pathway might be a common pathological mechanism underlying cerebellar ataxia in mouse and human.

Interestingly, besides adult-onset Purkinje cell loss, the *Mwk* mutation in TRPC3 causes impairments in Purkinje cell function and dendritic arborization during cerebellar development (8,10). In contrast, no dendritic phenotype and a milder form of ataxia have been observed in the *Trpc3* knockout mice (6,11). Hence, it is the sustained activation of TRPC3 that provides an important regulatory effect on dendritic growth in cerebellar Purkinje cells. This model is consistent with the observation that overexpression of SCA14-associated PKCγ mutants that fail to inhibit TRPC3 (12) causes impairments in the dendritic development of Purkinje cells (13). Similarly, related studies have shown the inhibition of Purkinje cell growth upon chronic activation of mGluR1 or PKC activation (14,15). However, the effector pathways that mediate the inhibition of dendritic growth downstream of activated TRPC3 have remained enigmatic.

Here, we report impairments in dendritic growth and synapse formation early on during Purkinje cell development in the *Mwk* cerebellum that are accompanied by alterations in key calcium signaling pathways. We also identify Purkinje cell-specific gene expression changes that result in abnormal lipid homeostasis in the mutant mice. Together, our study provides mechanistic insights into the TRPC3-dependent processes that link activated calcium signaling to lipid metabolism and the regulation of Purkinje cell development in the *Mwk* cerebellum.

## Results

### Dendritic and synaptic impairments in the developing *Mwk* cerebellum

**Figure 1.**
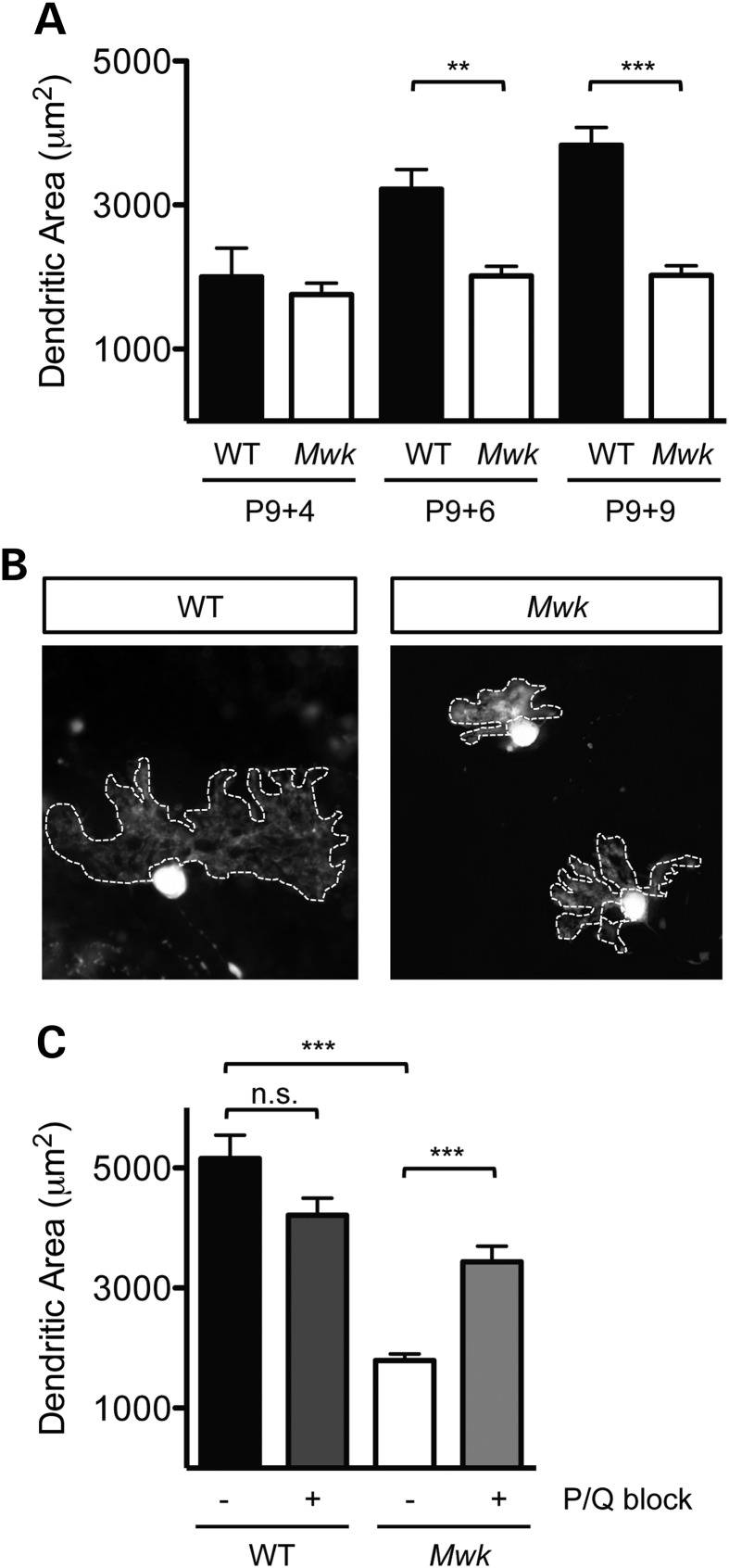
*Mwk* TRPC3 impairs Purkinje cell dendritic arborization. (**A**) Time course of Purkinje cell dendritic growth in organotypic slice cultures prepared from wild-type (WT) and mutant (*Mwk*) littermates. Cultures were fixed at the indicated days *in vitro* and stained with an anti-calbindin antibody to visualize dendritic morphology. Initially, *Mwk* dendritic arbors were indistinguishable from wild-type littermates but starting at around two weeks postnatally, *Mwk* Purkinje cells exhibited a significantly decreased size of their dendritic trees (*P* < 0.0001, ANOVA, *n* = 128). (**B**) Representative images of Purkinje cell dendritic morphology in organotypic slice cultures fixed at P9+DIV9 and immunostained for calbindin demonstrate the impaired arborization in *Mwk* Purkinje cells. The outlines of the dendritic arbors have been traced. (**C**) Size of the dendritic area in organotypic slice cultures (P9+DIV12) prepared from wild-type (WT) and mutant (*Mwk*) littermates, fixed and immunostained with an anti-calbindin antibody. Treatment with P/Q block significantly increased the dendritic area of the *Mwk* but not WT Purkinje cells (*P* < 0.0001, ANOVA, *n* = 218).

Previously, we demonstrated abnormal Purkinje cell dendritic morphology in 3-week-old *Mwk* mice (8). To investigate whether the mutant phenotype results from a failure of the dendritic tree to arborize or whether it is a consequence of atrophy following normal outgrowth, we carried out a time course experiment in organotypic cerebellar slice cultures of *Mwk* mice compared with their wild-type littermates. The initial development of the Purkinje cell dendritic tree was similar in wild-type and mutant mice based on the extent of the Purkinje cell dendritic area (Fig. 1A). However, starting from postnatal day 15 (P9+DIV6), impairments in arborization were observed in the *Mwk* Purkinje cells (Fig. 1A). Although wild-type Purkinje cells continued to grow their dendritic arbors, mutant Purkinje cells failed to further expand the area of their dendritic tree (Fig. 1A and B), suggesting that it is the late phase of dendritic outgrowth that is specifically impaired in *Mwk* Purkinje cells.

P/Q-type calcium channels have been shown to contribute to the inhibition of dendritic growth following activation of mGluR1 (11). Moreover, TRPC3 signaling in cardiac myocytes has been linked to activation of voltage-gated calcium channels (VGCCs) (16). These observations raised the question of whether VGCCs might contribute to the dendritic phenotype of Purkinje cells in the *Mwk* mouse. To assess whether inhibition of VGCCs could rescue the dendritic phenotype in *Mwk* Purkinje cells, we used a combination of ω-agatoxin IVA and ω-conotoxin MVIIC to inhibit P/Q-type calcium channels (P/Q block) (11). Organotypic slice cultures (P9) were treated for 12 days with P/Q block, starting at DIV2. As seen previously, dendritic arborization of *Mwk* Purkinje cells was significantly impaired compared with wild-type cells (Fig. 1C). Interestingly, inhibition of P/Q-type calcium channels significantly increased the dendritic area of *Mwk* Purkinje cells when compared with untreated *Mwk* cultures by 50% (4457 ± 316 μm^2^ versus 2176 ± 135.2 μm^2^) (Fig. 1C). Treatment of wild-type slices with P/Q block had no significant effect (Fig. 1C) (11). Together, these results suggest that P/Q-type calcium channels are involved in the dendritic growth inhibition of Purkinje cells that is caused by mutant *Mwk* TRPC3.

**Figure 2.**
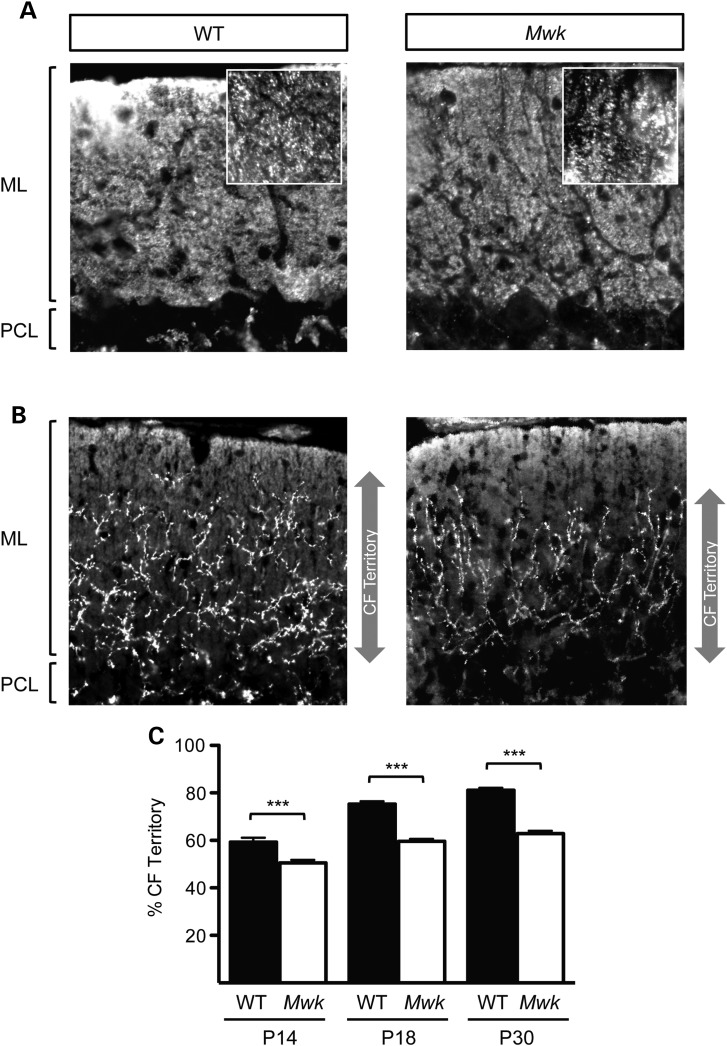
Altered distribution of climbing fiber (CF) terminals on Purkinje cell dendrites of *Mwk* mice. (**A**) Representative image of the distribution of VGLUT1-labeled parallel fiber (PF) terminals in the molecular layer (ML) of cerebellar sections from a 30-day-old *Mwk* mouse compared with a wild-type (WT) littermate. No difference in VGLUT1-positive puncta was noted (density VGLUT1 staining WT = 76.26 ± 7.093; *Mwk* = 74.54 ± 8.694; *P* = 0.7992; *n* = 3). (**B**) Representative image of the distribution of VGLUT2-labeledCF terminals along Purkinje cell (PC) dendrites in the ML of cerebellar sections from a 30-day-old *Mwk* mouse compared with a WT littermate. Arrows depict the extension of the CF territory. (**C**) At all timepoints tested (P14, P18, P30), *Mwk* mice show a significant reduction in the CF territory (*P* < 0.001, ANOVA, *n* = 126).

We next examined whether the dendritic changes observed in the *Mwk* mice were accompanied by changes in the placement of excitatory synaptic terminals onto Purkinje cell dendritic trees. The distal part of the Purkinje cell dendritic tree receives excitatory input from the parallel fibers (PFs) of the granule cells, whereas the proximal portion of the dendritic tree is innervated by inputs from climbing fibers (CFs). The innervation areas of PFs and CFs are intimately linked, and stabilization of PF synapses restricts the innervation territory of CFs on the Purkinje cell dendritic tree and vice versa (17,18). First, we investigated the PF innervation pattern of wild-type and *Mwk* Purkinje cells by their immunoreactivity to the vesicular glutamate transporter VGLUT1. We found no detectable differences in the immunostaining pattern for VGLUT1 in cerebellar sections from *Mwk* mice compared with their wild-type littermates (density VGLUT1 staining WT = 76.26 ± 7.093; *Mwk* = 74.54 ± 8.694; *P* = 0.7992; *n* = 3) (Fig. 2A). Both wild-type and mutant cerebellar sections exhibited strong VGLUT1-positive puncta representing PF inputs throughout the molecular layer. To determine the spatial distribution of CF terminals on Purkinje cell dendritic arbors, we carried out immunostainings for VGLUT2. In adult wild-type cerebellar sections, the distribution of VGLUT2 staining representing the extent of the CF territory extended to a mean relative height of 81 ± 0.9862% of the molecular layer (Fig. 2B and C), consistent with previous reports using wild-type mice (17,19,20). In contrast, in cerebellar sections of adult *Mwk* mice, the mean relative height was reduced to 63 ± 1.108%. We also assessed the extension of the CF territory in cerebellar sections of younger animals. A significant reduction in CF arborization along the Purkinje cell dendrites was observed in the mutants as early as postnatal day 14 (P14) (Fig. 2C), concurrent with the observed dendritic abnormalities (Fig. 1A). Together, these results suggest that *Mwk* mice exhibit profound dendritic and synaptic impairments during development as well as in adulthood.

### Altered calcium signaling pathways in the developing *Mwk* cerebellum

**Figure 3.**
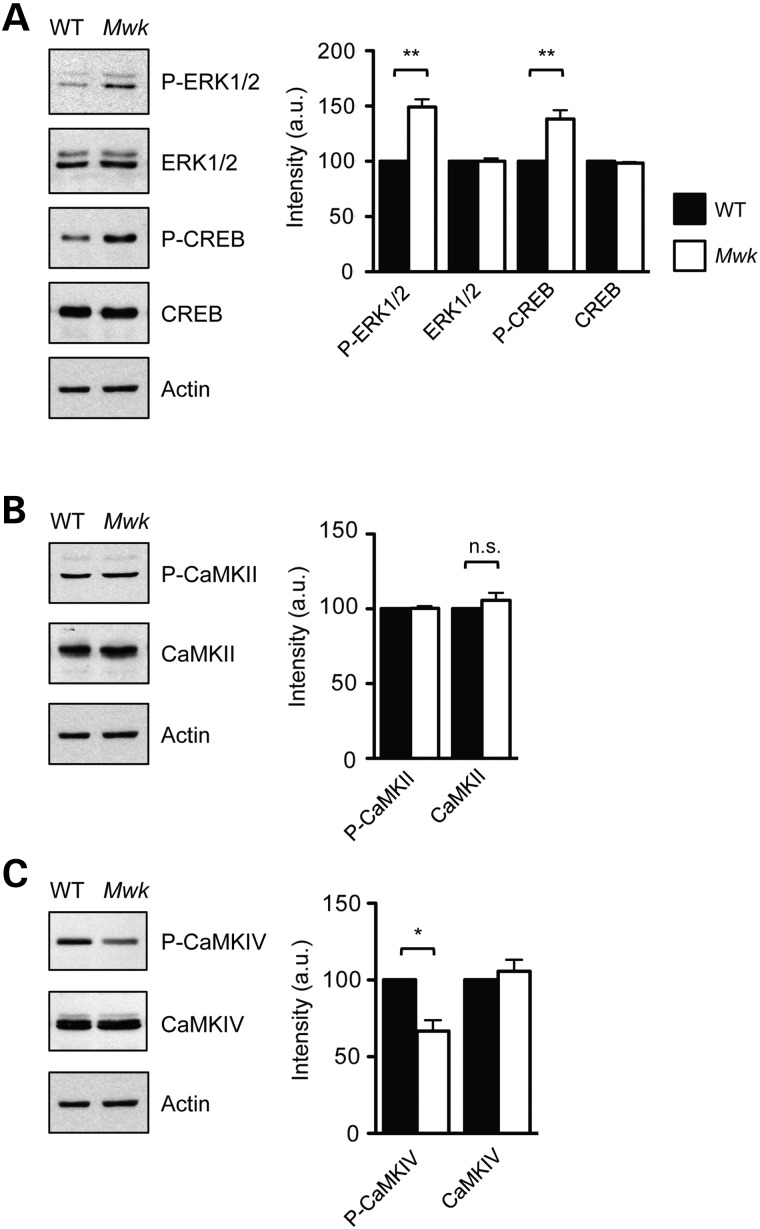
Activation of key calcium signaling pathways in *Mwk* cerebellum. Cerebellar extracts from 3-week-old mutant *Mwk* mice and wild-type (WT) littermates were subjected to immunoblotting for calcium-responsive proteins. (**A**) Phosphorylated ERK1/2 and phosphorylated CREB (Ser133) levels were increased in the *Mwk* cerebellum. (**B**) Levels of phosphorylated CaMKII remained unchanged in *Mw*k cerebellum. (**C**) Levels of phosphorylated CaMKIV were reduced in *Mwk* cerebellum. Actin levels are included as loading control. Representative blots are shown for 3-week-old animals. Densitometry quantifications are shown (right panels). Statistical significance was determined by one-way ANOVA (*n* = 3). *P< 0.05; **P< 0.01.

Having identified these dendritic and synaptic abnormalities, we next investigated the mechanism underlying *Mw*k TRPC3-mediated impairments in Purkinje cell development. As the *Mwk* phenotype results from a gain-of-function mutation in the calcium-permeable TRPC3 channel (8) and we have also shown that VGCCs are involved (Fig. 1C), we asked which calcium-sensitive downstream signaling pathways might be affected in the developing *Mwk* cerebellum. We prepared cerebellar extracts from *Mwk* mice and their wild-type littermates at several time points during postnatal development (P15–P46), followed by immunoblotting for key proteins of neuronal calcium signaling. We found that several calcium-sensitive signaling molecules were altered in the mutant cerebellum compared with wild-type controls. Changes were observed as early as 2 weeks postnatally. The phosphorylation status and thus the activity levels of both the extracellular-regulated kinases 1/2 (ERK1/2) and the transcription factor cAMP response element-binding protein (CREB) were increased in *Mwk* cerebellum consistent with activated calcium signaling (Fig. 3A). Levels of phosphorylated calcium/calmodulin-dependent protein kinase type II (CaMKII) were unchanged in *Mwk* cerebellum at all ages studied (Fig. 3B). In contrast, phosphorylation of calcium/calmodulin-dependent protein kinase type IV (CaMKIV) was reduced in the *Mwk* cerebellum (Fig. 3C). Noteworthy, phosphorylated CaMKIV is predominantly present in Purkinje cells of the cerebellum (Supplementary Material, Fig. S1). Hence, the changes in phosphorylated CaMKIV that were observed in total cerebellar extracts reflect specific changes in the Purkinje cells. Together, these findings demonstrate alterations in important calcium-sensitive signaling kinases and downstream transcription factors in the developing *Mwk* cerebellum.

### *Mwk* Purkinje cells display significant gene expression changes

**Figure 4.**
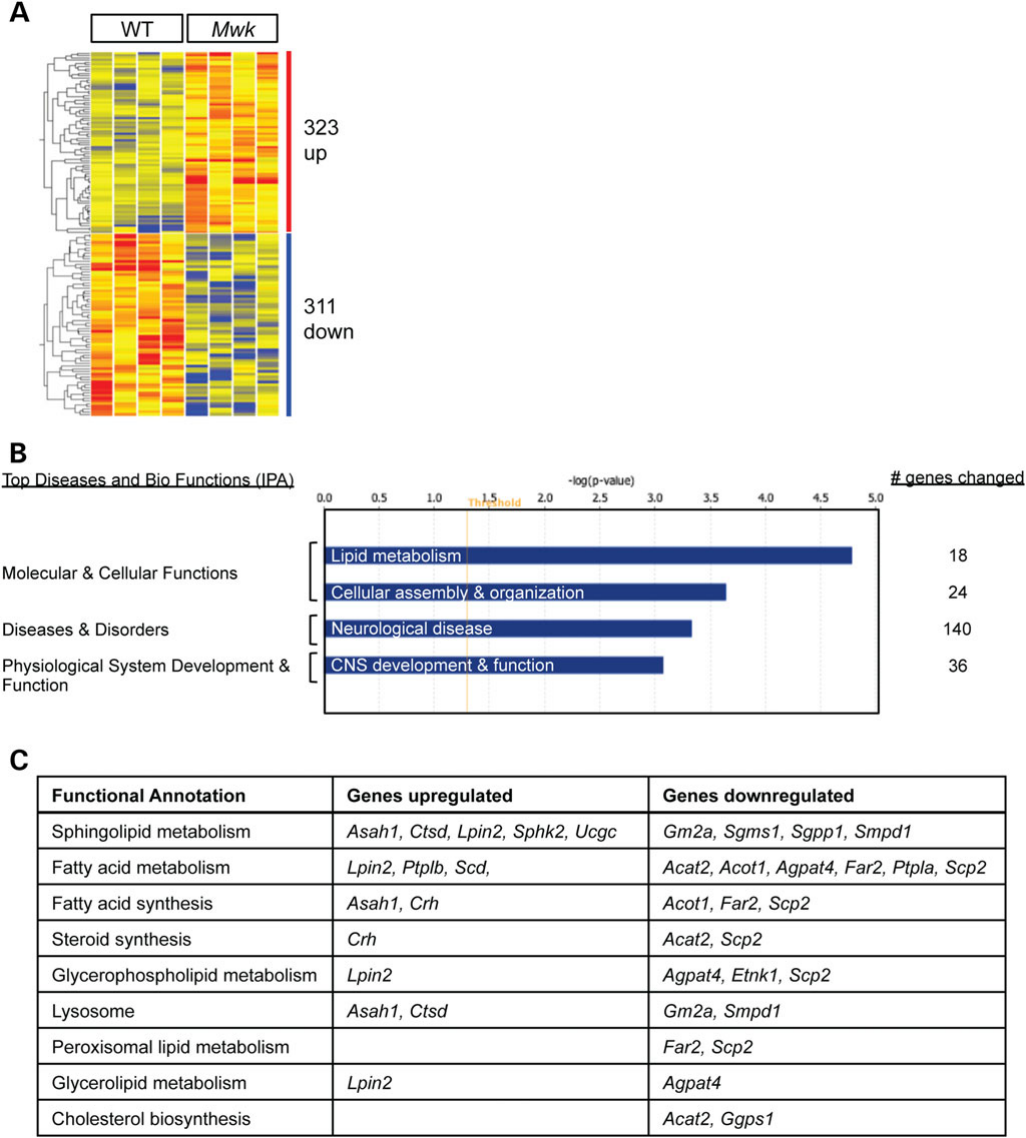
Gene expression changes in *Mwk* Purkinje cells. (**A**) Heat map shows statistically significant changes in the expression of 634 genes between WT and *Mwk* Purkinje cells at P18 (*P* ≤ 0.05). Differentially expressed genes are listed in Supplementary Material, Table S1. Each column represents one of four RNA samples/biological replicates for each genotype. (**B**) Significant enrichment in genes differentially expressed in *Mwk* Purkinje cells in IPA top functions and disease categories. The number of changed genes is indicated. (**C**) Functional annotation of differentially expressed genes associated with lipid metabolism that are listed in Table 1. Only categories with two or more genes are listed. Annotations were made using a combination of pathway analysis tools including IPA, Kyoto Encyclopedia of Genes and Genomes (KEGG) and SuperPaths (http://www.genecards.org/info.shtml#pathways_interactions).

Little is known about the specific targets regulated by calcium-mediated signaling pathways that control Purkinje cell development. On the basis of our findings, the *Mwk* mouse represents an ideal system to investigate the intrinsic factors that link altered calcium signaling and Purkinje cell development. To identify gene expression changes specifically in *Mwk* Purkinje cells, we carried out laser capture microdissection (LCM) of Purkinje cells, followed by RNA isolation and microarray analysis using the Affymetrix Mouse Gene 1.0 ST array. This was done in 18-day-old *Mwk* mice and their wild-type littermates, thus around the critical period and the onset of molecular and behavioral changes in the mutant mice. Samples were obtained from four sets of *Mwk* and wild-type littermates, and 1000 Purkinje cells were collected per cerebellum. Applying a stringent detection threshold (1.5-fold change) and cut-off *P*-value of ≤0.5, we identified statistically significant changes in the expression levels of 634 genes between wild-type and *Mwk* Purkinje cells. Fifty-one percent (323) of genes were upregulated and 49% (311) of genes were downregulated (Fig. 4A and Supplementary Material, Table S1). The results were reproducible among the four biological replicates (Fig. 4A).

To verify the microarray results, we selected several genes that showed significant expression changes between *Mwk* and wild-type Purkinje cells for independent validation by quantitative reverse transcription polymerase chain reaction (qRT-PCR) using LCM-isolated Purkinje cell RNA from an independent set of four *Mwk* mice and their wild-type littermates at P18. We detected similar changes in the expression levels of these genes by qRT-PCR as by microarray (Supplementary Material, Fig. S2 and Table S2). We also investigated the expression levels of these genes at earlier timepoints to determine how early we could detect changes in the mutant cerebellum. We found three of the selected genes (*Ipo5*, *Opn3*, *Sv2c*) upregulated in *Mwk* Purkinje cells as early as P11. Others were downregulated in the mutant Purkinje cells from P14 (*Stk17b*, *Car2*) (Supplementary Material, Table S2). Consistent with the expression changes observed by microarray and qRT-PCR, differences in mRNA expression levels between *Mwk* Purkinje cells and wild-type littermates were also detected by *in situ* hybridization (ISH) (Supplementary Material, Fig. S3). Together, these data show that significant gene transcription changes occur in *Mwk* mice early on in Purkinje cell development, concurrent with the observed dendritic and synaptic abnormalities.

### *Mwk* Purkinje cells exhibit gene expression changes linked to lipid metabolism

To gain insights into the potential molecular pathways affected in *Mwk* Purkinje cells, we next analyzed the differentially expressed genes using Ingenuity® Pathway Analysis (IPA). This analysis revealed several biological pathways and functions that were significantly enriched in dysregulated genes of the *Mwk* cerebellum including cellular assembly and organization (*P* = 2.28E-04–4.38E-02) and nervous system development and function (*P* = 8.40E-04–4.21E-02), consistent with the developmental phenotype of the mice (Fig. 4B, Supplementary Material, Tables S3 and S4). Moreover, genes associated with neurological disease were significantly affected (*P* = 4.65E-04–4.34E-02) (Fig. 4B). Notably, included in this category were movement disorders (50 gene changes; *P* = 1.70E-03) and ataxia (13 gene changes; *P* = 1.59E-02) (Supplementary Material, Table S5).

**Figure 5.**
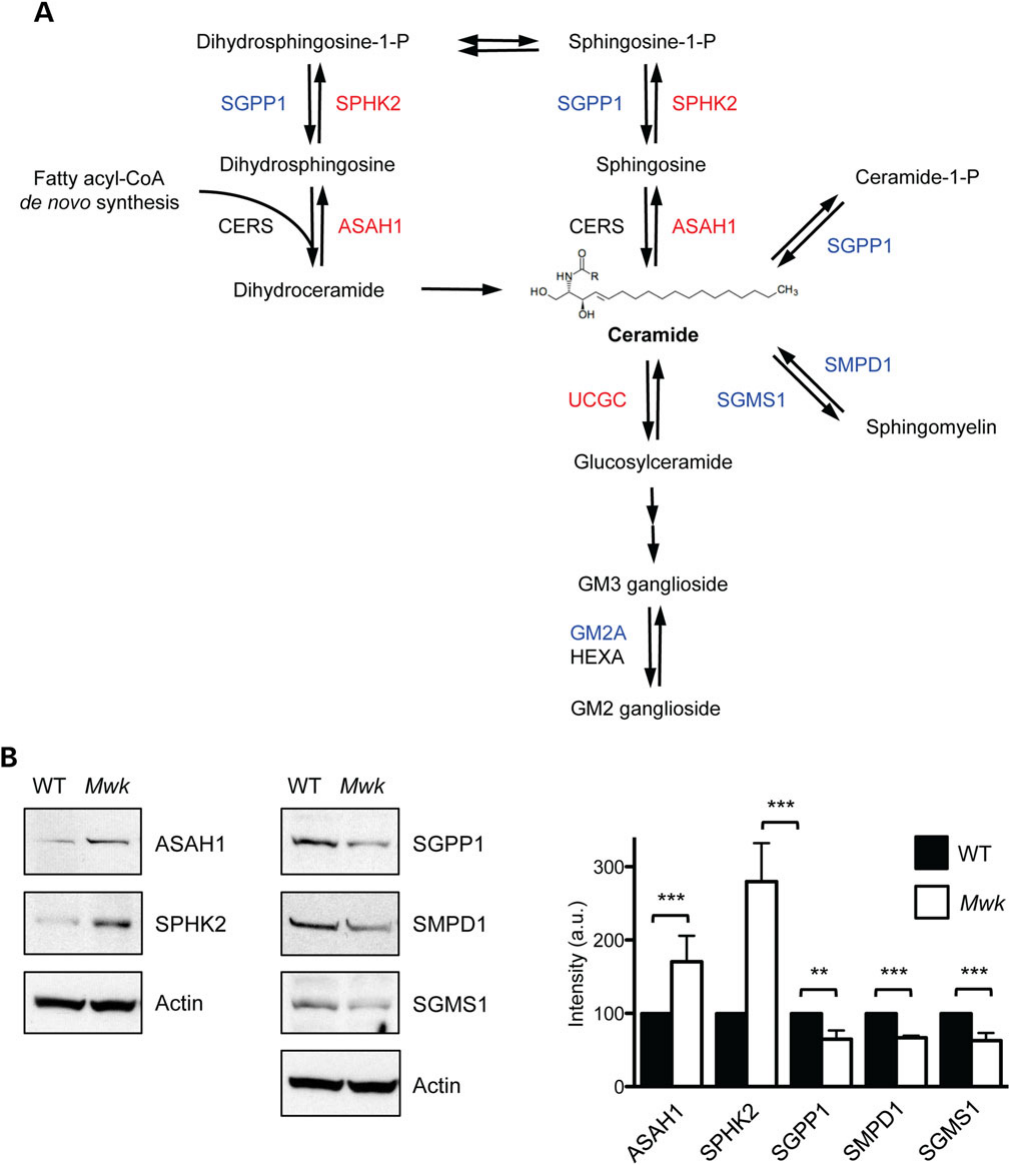
Protein expression changes in enzymes involved in ceramide homeostasis. (**A**) Schematic diagram of mammalian ceramide biosynthesis. Differentially expressed enzymes in *Mwk* Purkinje cells are indicated in red (upregulated) and blue (downregulated). (**B**) Cerebellar extracts from 3-week-old mutant *Mwk* mice and WT littermates were subjected to immunoblotting for lipid metabolism enzymes. ASAH1 and SPHK2 levels were increased in the *Mwk* cerebellum (left panel), whereas levels of SGPP1, SMPD1 and SGMS1 were reduced in *Mwk* cerebellum (middle panel). Actin levels are included as loading control. Densitometry quantifications are shown (right panel). Statistical significance was determined by one-way ANOVA (*n* = 3). **P< 0.01; ***P< 0.001.

Interestingly, the top enriched category for genes altered in *Mwk* Purkinje cells was lipid metabolism, and one of the top altered canonical pathways in *Mwk* Purkinje cells was sphingolipid metabolism (Fig. 4B, Supplementary Material, Tables S6 and S7). We identified 27 differentially expressed genes that are associated with lipid metabolism, of which 10 were upregulated in *Mwk* Purkinje cells and 17 were downregulated (Table 1). We classified these genes further into sub-categories of lipid metabolism using a combination of pathway analysis tools. Consistent with the results described above, we found that a large number of affected genes encode proteins that are key enzymes in sphingolipid metabolism, particularly the synthesis of ceramide (Fig. 4C). Notably, we observed reciprocal expression changes in the enzymes sphingosine kinase 2 (SPHK2) and sphingosine-1-phosphate phosphatase 1 (SGPP1) as well as changes in the expression of acid ceramidase (ASAH1), sphingomyelin synthase 1 (SGMS1) and sphingomyelin phosphodiesterase 1 (SMPD1), suggesting a coordinated deregulation of ceramide biosynthesis (Fig. 5A). To validate these gene expression changes at the protein level, we prepared cerebellar extracts from three-week-old *Mwk* mice and their wild-type littermates and subjected these to immunoblotting. Consistent with the observed microarray changes, we found the protein levels of ASAH1 and SPHK2 upregulated in the *Mwk* cerebellum (Fig. 5B). Conversely, SGPP1, SMPD1 and SGMS1 levels were reduced in the mutant cerebellum (Fig. 5B). Together, these enzyme expression changes point towards a reduction in the pool of available ceramide within the *Mw*k Purkinje cells. In addition, we observed expression changes in UDP-glucose ceramide glycosyltransferase (UCGC) and G_M2_ ganglioside activator protein (GM2A), two enzymes that are involved in the synthesis and degradation of complex glycosphingolipids that are linked to the ceramide pathway (Fig. 5A).

**Table 1.** Significant expression changes (≥1.5-fold) in *Mwk* Purkinje cells compared with wild-type littermates in genes associated with lipid metabolism (*P* ≤ 0.05)

Gene symbol	Gene product	Fold change
*Ctsd*	Cathepsin D	5.7
*Ptplb*	Protein tyrosine phosphatase-like (proline instead of catalytic arginine), member b	4.3
*Prdx6*	Peroxiredoxin 6	3.5
*Scd*	Stearoyl-Coenzyme A desaturase 1	2.8
*Asah1*	*N*-acylsphingosine amidohydrolase 1 (acid ceramidase)	2.4
*Ugcg*	UDP-glucose ceramide glucosyltransferase	2.0
*Dab1*	Disabled homolog 1	1.8
*Lpin2*	Lipin 2	1.7
*Sphk2*	Sphingosine kinase 2	1.5
*Crh*	Corticotropin releasing hormone	1.5
*Scp2*	Sterol carrier protein 2	−5.7
*Ptpla*	Protein tyrosine phosphatase-like (proline instead of catalytic arginine), member a	−4.9
*Gdpd1*	Glycerophosphodiester phosphodiesterase domain containing 1	−3.2
*Acot1*	Acyl-CoA thioesterase 1	−3.1
*Far2*	Fatty acyl CoA reductase 2	−2.8
*Fgf7*	Fibroblast growth factor 7	−2.5
*Sgpp1*	Sphingosine-1 phosphate phosphatase 1	−2.2
*Smpd1*	Sphingomyelin phosphodiesterase 1, acid lysosomal	−1.9
*Ggps1*	Geranylgeranyl diphosphate synthase	−1.9
*Etnk1*	Ethanolamine kinase 1	−1.8
*Osbpl1a*	Oxysterol binding protein-like 1A	−1.8
*Ncoa4*	Nuclear receptor coactivator 4	−1.6
*Gm2a*	GM2 ganglioside activator protein	−1.5
*Sgms1*	Sphingomyelin synthase 1	−1.5
*Agpat4*	1-acylglycerol-3-phosphate O-acyltransferase 4	−1.5
*Gfra2*	Glial cell line derived neurotrophic family receptor alpha 2	−1.5
*Acat2*	Acetyl-Coenzyme A acetyltransferase 2	−1.5

**Figure 6.**
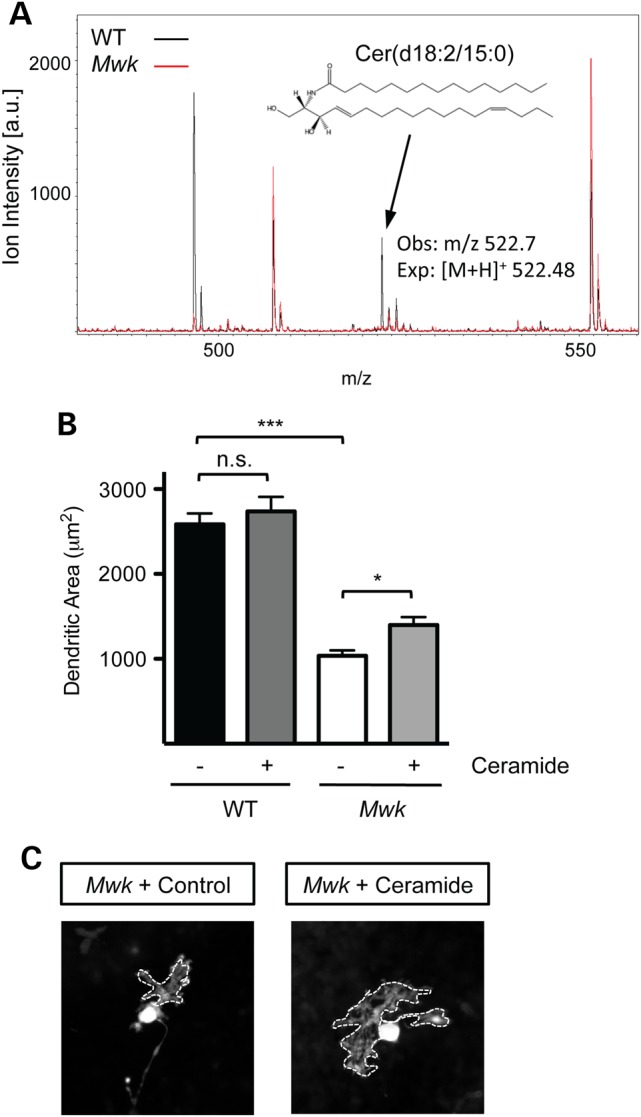
Altered ceramide homeostasis in *Mwk* Purkinje cells. (**A**) MALDI-TOF mass spectrometry analysis of cerebellar lipid extracts from *Mwk* mice and wild-type (WT) littermates at P18. Following mass spectrometry, spectrum peaks were annotated using the METLIN metabolite database (http://metlin.scripps.edu/index.php). Mass spectrometry spectra overlay of lipid profiles from *Mwk* (red) and WT cerebellum (black) show a reduction of ceramide Cer(d18:2/15:0), depicted by an arrow (observed *m/z* 522.7; expected [M+H]^+^ 522.5). The graph is a representative of the results obtained from three independent biological replicates. (**B**) The size of the dendritic area in organotypic slice cultures (P9+DIV12) prepared from wild-type (WT) and mutant (*Mwk*) littermates, fixed and immunostained with an anti-calbindin antibody. Treatment with C6-ceramide (Cer) but not inactive control (−) significantly increased the dendritic area of the *Mwk* but not WT Purkinje cells (*P* < 0.05, ANOVA, *n* = 202). (**C**) Representative images of Purkinje cell dendritic morphology in organotypic slice cultures fixed at P9+DIV12 and immunostained for calbindin demonstrate the increased arborization in *Mwk* Purkinje cells treated with C6-ceramide compared with inactive control. The outlines of the dendritic arbors have been traced.

Having identified that the expression levels of enzymes involved in lipid metabolism are significantly changed in the *Mwk* mutant, we next investigated whether levels of lipids were changed as a consequence in the *Mwk* cerebellum. We carried out lipid analysis by mass spectrometry in the cerebella of three *Mwk* mice and their wild-type littermates at P18. Consistent with the expression changes observed in *Mwk* Purkinje cells, we identified alterations of lipid levels in the mutant cerebellum including a reduction in distinct ceramides (Fig. 6A). We also observed changes in other lipids in the *Mwk* cerebellum, including fatty acyls and sterol lipids (data not shown). Interestingly, changes in lipid metabolism were not only evident in our analysis of the *Mwk* cerebellum, but serum levels of free fatty acids, triglycerides and cholesterol have also been found to be significantly changed in the *Mwk* mice in the EuroPhenome mouse phenotyping project (Supplementary Material, Fig. S4) (21).

We next tested whether treatment of *Mwk* organotypic slice cultures with ceramide might alleviate the dendritic phenotype observed in the mutant Purkinje cells. Sphingolipids have previously been shown to be important for Purkinje cell dendritic differentiation (22,23). Moreover, treatment of organotypic cerebellar cultures with sphingomyelinase was demonstrated to enhance dendritic branching complexity of Purkinje cells (23). In mice sphingomyelinase is encoded by the gene *Smpd1*, the expression of which is downregulated in *Mwk* cerebellum (Fig. 5A and B, Table 1). We prepared organotypic cerebellar slice cultures from *Mwk* mice and their wild-type littermates at P9 and treated these for 12 days *in vitro* with C6-ceramide or its inactive form dihydroceramide C6 as control. We found that treatment of the *Mw*k cerebellar slices with C6-ceramide significantly improved Purkinje cell dendritic outgrowth compared with control (Fig. 6B and C). Together, these findings suggest that the *Mwk* gain-of-function mutation in TRPC3 results in aberrant calcium signaling and gene expression changes in Purkinje cells that lead to alterations in lipid homeostasis and impairments in dendritic growth.

## Discussion

In this study, we describe early dendritic and synaptic impairments and altered calcium-activated signaling pathways during Purkinje cell development in the *Mwk* mouse, an ataxic mouse model in which we previously identified a novel gain-of-function mutation in the non-selective cation channel TRPC3 (8). Combining Purkinje cell-specific transcriptomic and cerebellar lipid analysis, this study provides the first mechanistic insights into the molecular pathways underlying the *Mwk* phenotype. Together, our findings support the model that activated TRPC3 leads to aberrant calcium signaling in Purkinje cells and subsequent gene expression changes that result in altered lipid homeostasis and consequently abnormal dendritic development.

Purkinje cells form one of the most elaborate dendritic arbors among all neurons in the brain. Although the morphological stages of Purkinje cell dendritic development have extensively been characterized, relatively little is known about the molecular mechanisms that underlie these processes (24–26). Previous work has demonstrated that chronic activation of Purkinje cell post-synaptic signaling including mGlur1 (14), PKC (15,27) and TRPC3 (8) causes inhibition of dendritic growth. Consistent with this hypothesis, Purkinje cells in the *lurcher* mouse mutant, which harbors a gain-of-function mutation in the δ2 glutamate receptor (GluD2) resulting in chronic depolarization, also exhibit impaired dendritic growth (28). These signaling events may provide a negative feedback mechanism for limiting the size of the dendritic tree once Purkinje cells have established appropriate synaptic contacts with the parallel fibers in the developing cerebellum. However, the downstream effector pathways mediating this inhibitory effect remain elusive. Calcium signaling is thought to regulate dendritic growth and patterning through distinct mechanisms that are dependent on calcium entry (local/global), timing (acute/chronic), neuronal cell type and developmental stage (29,30). Our study points to specific calcium-sensitive pathways that may restrict dendritic growth in Purkinje cells upon sustained TRPC3 activation and chronic Purkinje cell depolarization. Interestingly, we found that CaMKIV is hypophosphorylated in the *Mwk* cerebellum compared with wild-type controls (Fig. 3B). Our findings of ataxia, impaired dendritic Purkinje cell growth and reduced CaMKIV activity in *Mwk* mutants are consistent with the phenotype observed in CaMKIV-deficient mice. The latter also display cerebellar ataxia and reduced dendritic arborization of Purkinje cells (31). CaMKIV is predominantly nuclear and is thought to regulate gene transcription either directly or through co-factors (32). Notably, one of the candidate transcription factors regulated by CaMKIV is RORα, an orphan member of the nuclear hormone-receptor superfamily (33,34). RORα is abundantly expressed in Purkinje cells (35) and strongly implicated in Purkinje cell development (36) as well as maintenance (37). Moreover, RORα has been linked to gene expression regulation that controls lipid metabolism (36). We have performed an initial bioinformatics-based analysis to identify transcription factor enrichment among the genes that are differentially expressed in *Mwk* Purkinje cells, which identified RORα among others (Supplementary Material, Table S8). In view of our findings, these observations raise the interesting possibility that RORα might link TRPC3 and CaMKIV signaling to gene expression changes in the *Mwk* cerebellum. Interestingly, *staggerer* mice with reduced RORα expression display decreases in triglycerides and cholesterol levels similar to the changes we observed in the *Mwk* mice (38).

Deficiencies in sphingolipid metabolism have been implicated in a range of neurological diseases, many of which are associated with cerebellar ataxia. Examples include autosomal-recessive cerebellar spasticity, which was recently been found to be caused by mutations in the *GBA2* gene (39,40) and many lysosomal storage disorders (41). Notably, mice that are deficient in enzymes that we found to be downregulated in the *Mwk* cerebellum also display cerebellar dysfunction including *Scp2* knockout mice (42), *Smpd1*-deficient mice, a model of Niemann-Pick disease (43), and *Gm2a* knockout mice, a model of G_M2_-gangliosidosis (44). Moreover, a deficiency of ceramide biosynthesis caused by mutations in the *Lass1* gene encoding ceramide synthase 1 (CerS1) (Fig. 5A) has been shown to cause cerebellar ataxia and Purkinje cell degeneration (45). Interestingly, Purkinje cell arborization is also greatly reduced in the *Lass1* mutants (45). The overlap in affected proteins and cerebellar phenotypes between the *Mwk* mouse and models of lysosomal storage diseases might point to converging pathological pathways underlying the cerebellar dysfunction in these models. It will be interesting to determine in future studies whether lipid-related pathways are also affected in other genetic forms of cerebellar ataxia and contribute to pathology more generally. This might be particularly relevant for those cerebellar ataxias that are linked to TRPC3 signaling such as SCA1, SCA14 and SCA15 (1) (reviewed in (7)).

Although long implicated in neuronal differentiation, the precise role of sphingolipids in dendritic development is unclear. Membrane traffic is required to supply growing dendrites with building material and hence, abnormalities in the production of required lipids might impede this process. It is also conceivable that ceramide and other sphingolipids control dendritic differentiation through their role in the formation of lipid rafts and the organization and activation of signaling receptors (46). Finally, ceramides have been demonstrated to act as a second messenger in signal transduction where they participate in growth inhibition, induction of differentiation and programmed cell death (47).

Although this study is focused on altered lipid homeostasis in the *Mwk* cerebellum, changes in other pathways are likely to contribute to the *Mwk* phenotype. The observed gene expression changes in several genes associated with vesicle fusion and exocytosis including *Sv2c* (Supplementary Material, Figs S2 and S3) point towards alterations in the secretory pathway in the *Mwk* cerebellum, which has a known role in dendritic arborization in many neuronal cell types (48). Of interest are also gene changes that have been observed in other ataxic mouse models such as the downregulation of the *Car2* gene (Supplementary Material, Fig. S2), encoding carbonic anhydrase II, which has also been reported in a mouse model of spinocerebellar ataxia type 7 (SCA7) (49). Moreover, protein levels of carbonic anhydrase II are decreased in the ataxic *lurcher* mouse mutant (50). Carbonic anhydrase II is highly and specifically expressed in developing Purkinje cells and has been postulated to be involved in differentiation and synaptogenesis of Purkinje cells (50). These findings highlight another potential converging pathological mechanism in the *Mwk* mouse and other models of cerebellar ataxia that will be interesting to explore further.

Our study identifies molecular alterations in the *Mwk* cerebellum that are common to other mouse models of cerebellar ataxia. Future studies will reveal whether these alterations pinpoint to converging pathways that could be targeted to modulate or monitor the pathogenesis of genetically different cerebellar diseases.

## Materials and Methods

### Animals and reagents

The generation of *Mwk* mice has been described elsewhere (8). The *Mwk* colony was maintained by backcrossing to C3H/HeH. All animal work was approved by the University of Oxford Ethics Panel and in accordance with UK Home Office regulations. All reagents were purchased from Sigma unless otherwise noted.

### Organotypic slice cultures

Organotypic slice cultures were prepared as previously described (8). The medium was changed every 2–3 days. For experiments using P/Q block, 100 nM ω-agatoxin IVA (Bachem) and 1 μM ω-conotoxin MVIIC (Bachem) were added from day 2 onwards (11). For ceramide treatment, 50 μM of C6-ceramide (*N*-hexanoyl-d-sphingosine) or its inactive form dihydroceramide C6 were added from day 2 onwards. At days-*in vitro* (DIV) 5–12, slice cultures were fixed in 4% paraformaldehyde followed by brief methanol permeabilization. Slices were blocked in PBS containing 0.3% Triton-X and 3% normal goat serum for 1 h at room temperature and incubated with rabbit anti-calbindin D-28K (Swant; 1:5000) for two nights at 4°C, followed by incubation with Alexa Fluor 594 goat anti-rabbit antibody (Invitrogen; 1:2000). For the quantification of Purkinje cell dendritic arbors, the size of the dendritic area was measured by tracing the outline of the dendritic tree using Axiovision 4.3 software (Zeiss) in a blinded manner. Cells were acquired from three independent experiments. Data were analyzed using GraphPad Prism. All data are represented as mean ± SEM. Statistical significance was assessed by one-way ANOVA followed by Bonferroni's multiple comparison test (*P< 0.05; **P< 0.01; ***P< 0.001).

### Biochemical analysis

Three-week-old mice were sacrificed and cerebella were dissected out and kept on ice. Protein extracts were prepared by 10-s sonication in an RIPA buffer (50 mm Tris pH 8, 150 mm sodium chloride, 1% Nonidet P40, 0.5% sodium deoxycholate, 0.1% sodium dodecyl sulfate (SDS), 100 μM sodium vanadate, 10 mm sodium fluoride, 1× Complete Protease Inhibitors [Roche]) followed by 10-min incubation on ice and 15-min centrifugation at 14 000*g* at 4°C. Fifty micrograms of protein extracts were analyzed by SDS–polyacrylamide gel electrophoresis and immunoblotting. The following antibodies were used: p44/42 MAPK (Erk1/2) (Cell Signaling; 1:1000), phospho-p44/42 MAPK (Erk1/2) (Tyr202/Tyr204) (Cell Signaling; 1:1000), CaMKIV (Abcam; 1:1000), p-CaMKIV (Thr196) (Santa Cruz; 1:1000), CaMKII (Cell Signaling, 1:500), phospho-CaMKII (Cell Signaling, 1:1000), CREB (Cell Signaling, 1:500), phospho-CREB (Ser133) (Cell Signaling, 1:1000), ASAH1 (Santa Cruz, 1:200), SPHK2 (Novus, 1:500), SGPP1 (Novus, 1:1000), SMPD1 (Santa Cruz, 1:200), SGMS1 (Santa Cruz, 1:200), Actin (Abcam, 1:1000). Antibody binding was detected by enhanced chemoluminescence (ECL, GE Healthcare). The intensity of the bands was quantified using ImageJ software (NIH). Data were normalized to actin levels and wild-type bands and analyzed using GraphPad Prism. All data are represented as mean ± SEM. Statistical significance was assessed by one-way ANOVA (*P< 0.05; **P< 0.01; ***P< 0.001).

### Immunohistochemistry

Freshly dissected cerebella from *Mwk* mice and wild-type littermates were fixed overnight in 4% paraformaldehyde, cryoprotected in 30% sucrose, embedded in OCT compound (TissueTek) and frozen. 10-μm sagittal sections were incubated for 30 min in 0.1 M glycine buffer (pH7.4), blocked for 1 h (10% goat serum, 0.3% Triton-X100 in PBS) and incubated with primary antibodies in block solution overnight at 4°C. The following antibodies were used: VGLUT1 (Synaptic Systems, 1:500), VGLUT2 (Synaptic Systems; 1:200), p-CaMKIV (Thr196) (Santa Cruz; 1:50) and Calbindin D28k (Synaptic Systems; 1:200). Sections were incubated with Alexa Fluor-labeled secondary antibodies (Invitrogen; 1:2000) for 3 h at room temperature before being mounted using DAPI-containing Vectashield medium (Vector Labs).

### LCM and RNA preparation

Freshly dissected cerebella from *Mwk* mice and wild-type littermates were embedded in OCT compound (TissueTek) and stored at −80°C until sectioning. 10-μm sagittal midline sections were cut and mounted onto PEN membrane-coated slides (Carl Zeiss), before fixation in a graded series of ice-cold ethanol solutions (95, 75 and 50%) (containing ProtectRNATM RNase inhibitor (Sigma)) for 30 s each, and staining for 1 min with 1% cresyl violet acetate. Sections were rinsed in 50% ethanol solution and dehydrated in 75, 95 and 100% ethanol solutions for 30 s each, and dehydrated twice in xylene for 30 s. Slides were left to air-dry briefly before proceeding with the LCM. Purkinje cells were microdissected from all cerebellar lobes of the sections using the P.A.L.M. Micro Beam system in combination with Robo software (Zeiss) and directly catapulted into a tube cap containing RLT lysis buffer with β-mercaptoethanol (RNeasy Micro kit (Qiagen)). One thousand Purkinje cells were collected for microarray analysis and 300–500 cells were collected for qRT-PCR. Total RNA was extracted using the RNeasy Micro Kit (Qiagen) and RNA quality was assessed on a 2100 BioAnalyzer using the RNA 6000 Pico Assay (Agilent Technologies). All samples used for microarray and qRT-PCR analysis had an RNA Integrity Number (RIN) of ≥6.

### Microarray analysis

Fragmented and labeled cDNA was generated from 1 ng of total RNA using the Ovation Pico WTA System, the WT-Ovation Exon Module and the Encore Biotin Module (all NuGEN) according to manufacturer's instructions and hybridized to the Affymetrix Mouse Gene 1.0 ST Array. Chips were processed on an Affymetrix GeneChip Fluidics Station 450 and Scanner 3000.

Microarray data were normalized by Probe Logarithmic Intensity Error (PLIER) using GeneSpring GX11.0 (Agilent), and differentially expressed genes (fold change difference ≥1.5) were identified using a moderated *t*-test with a Benjamini and Hochberg multiple testing correction of 0.05. Differentially expressed genes were hierarchically clustered. Gene interaction networks and canonical pathways were analyzed using IPA (www.ingenuity.com). Further functional annotation was carried out using a combination of IPA, Kyoto Encyclopedia of Genes and Genomes (KEGG) and SuperPaths (http://www.genecards.org/info.shtml#pathways_interactions). Microarray data are available in the ArrayExpress database (www.ebi.ac.uk/arrayexpress) under accession number E-MTAB-3517.

### Lipid analysis by mass spectrometry

Freshly dissected cerebella from P18 *Mwk* mice and wild-type littermates were homogenized by 10-s sonication in an RIPA buffer (50 mm Tris pH 8, 150 mm sodium chloride, 1% Nonidet P40, 0.5% sodium deoxycholate). Subsequently, lipid material was extracted by methanol/chloroform (4:1). The aqueous and organic phases were isolated, dried down in vacuum and resuspended in acetone/methanol (2:1). The samples were then subjected to analysis by matrix-assisted laser desorption time-of-flight (MALDI-TOF) mass spectrometry (MS) using an Ultraflex II (Bruker Daltonics) mass spectrometer. Lipid profiles were analyzed using FlexAnalysis (Bruker Daltonics, v2.4) and annotated using the METLIN metabolite database (http://metlin.scripps.edu/index.php).

## Supplementary Material

Supplementary Material is available at *HMG* online.

*Conflict of Interest statement*. None declared.

## Funding

This work was supported by the UK Medical Research Council and the Royal Society. We thank the Wellcome Trust Integrative Physiology Initiative in Ion Channels and Diseases of Electrically Excitable Cells (OXION) for use of the microarray facility. E.B. is the recipient of a Research Fellowship from the Royal Society. B.M.K. was supported by the Biomedical Research Centre (NIHR), Oxford, and the Kennedy Trust Fund. Funding to pay the Open Access publication charges for this article was provided by the University of Oxford RCUK Open Access Block Grant.

## Supplementary Material

Supplementary Data
