# Supplementary material for: The mutant Moonwalker TRPC3 channel links calcium signaling to lipid metabolism in the developing cerebellum
Source: Hum Mol Genet. 2015 Apr 23;24(14):4114–25. doi: 10.1093/hmg/ddv150 (PMC4476454; doi:10.1093/hmg/ddv150)
Supplement: Supplementary Data [file supp_24_14_4114__index.html]

The mutant Moonwalker TRPC3 channel links calcium signaling to lipid metabolism in the developing cerebellum — The mutant Moonwalker TRPC3 channel links calcium signaling to lipid metabolism in the developing cerebellum — Supplementary Data 

# The mutant *Moonwalker* TRPC3 channel links calcium signaling to lipid metabolism in the developing cerebellum

## Supplementary Data

Supplementary Data

**Files in this Data Supplement:**

- Supplementary Data - Doc file
- Supplementary Table 1 - xlsx file
- Supplementary Table 3 - xls file
- Supplementary Table 4 - xls file
- Supplementary Table 5 - xls file
- Supplementary Table 6 - xls file
- Supplementary Table 7 - xls file
- Supplementary Table 8 - xlsx file
