# Supplementary material for: The mutant Moonwalker TRPC3 channel links calcium signaling to lipid metabolism in the developing cerebellum
Source: Hum Mol Genet. 2015 Apr 23;24(14):4114–25. doi: 10.1093/hmg/ddv150 (PMC4476454; doi:10.1093/hmg/ddv150)
Supplement: Supplementary Data [file supp_ddv150_ddv150supp.doc]

**Dulneva *et al.***

**The mutant *Moonwalker* TRPC3 channel links calcium signaling to lipid metabolism in the developing cerebellum**

**Supplemental Materials and Methods**

*Quantitative reverse transcription polymerase chain reaction (qRT-PCR)*

Total cerebellar RNA was reverse transcribed using the Superscript III First-Strand Synthesis System (Invitrogen) according to manufaturer’s instructions. Real-time PCR was performed on an ABI PRISM 7500 system (Applied Biosystems) using SYBR Green PCR Master Mix (Applied Biosystems). Three technical replicates per reaction were run. Data were analysed using StepOne software (Applied Biosystems) using standard ΔΔCt calculations. Calbindin levels were chosen as internal normalizing control as expression levels do not change between wildtype and *Mwk* mice.

The following primers were used:

| Primer |  | Sequence |
| --- | --- | --- |
| Calbindin | F  R | 5’ TGTGTGGGAAAGAGTTCAATAAGG 3’  5’ TCCAGCTCATTTTCATCTATGTATCC 3’ |
| Car2 | F  R | 5’ AAGATTGGACCTGCCTCACAAG 3’  5’ ACGCTTCCCCTTTGTTTTAATG 3’ |
| Cntn3 | F  R | 5’ TGGAAGCCGATCTGAACTAGTG 3’  5’ AAGCAACCACATACCCAAAACC 3’ |
| Ipo5 | F  R | 5’ GCGCAGTTTGGTGGAGATAAC 3’  5’ AGCCTCTGGAGCCTGAATGAC 3’ |
| Opn3 | F  R | 5’ TCGAATGCTTCGCTGTGTTG 3’  5’ CCATCAAAAAGCACATCTTTGC 3’ |
| Stk17b | F  R | 5’ CGGGAGGACAAGGAGAACATC 3’  5’ GGCAAGGAGTCATCGAATCG 3’ |
| Sv2c | F  R | 5’ TGCATGTCTGTCAACGGATTC 3’  5’ AGCAACCGACAGACGAGAAAG 3’ |

*In situ hybridization (ISH)*

ISH probes were generated by PCR from mouse cerebellar cDNA, cloned into pCR4-TOPO (Invitrogen) and digoxigenin (DIG, Roche)-labeled riboprobes were synthesized from linearized plasmid DNA using T7 and T3 RNA polymerases (Roche). The following primer sequences were used for riboprobe cloning (5’-to-3’): Ipo5F: CCAGAAGGAGCTGAGACTGC; Ipo5R: TAACCACGGGAAGGTACTGC, Sv2cF: TTCAGCCTACCAGTTCCACAG; Sv2cR: AATTCCGTACAGTTCGGTGC.

Cerebellar tissue samples were snap-frozen and 10-μm midline sagittal sections were cut and mounted onto Superfrost Plus slides (VWR). Probe hybridization, washing, and signal detection using an alkaline phosphatase-conjugated anti-DIG antibody (Roche) was carried out as previously described (1). Sections were developed in parallel for 12-48 h in all cases to prevent saturation of the expression signal.

**Supplemental Figures**


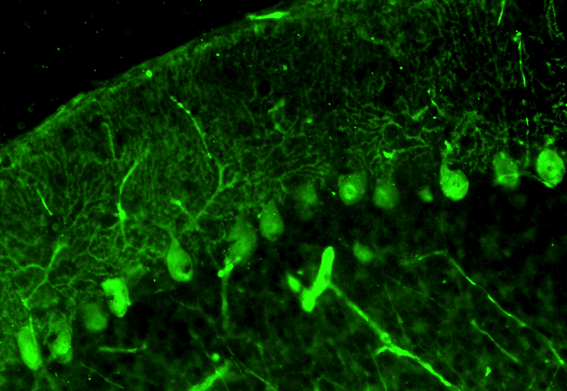

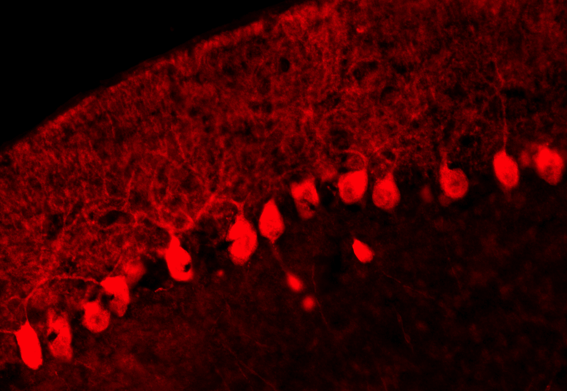


p-CaMKIV


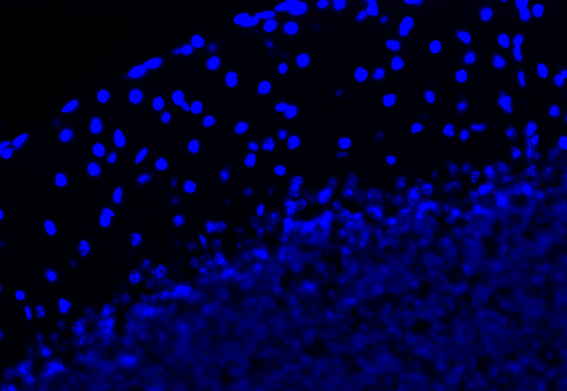


DAPI


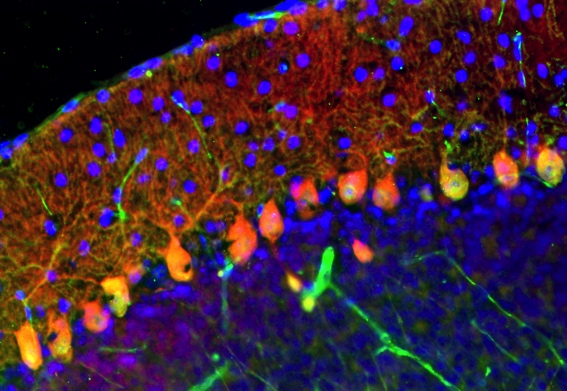


Merge

Calbindin

**Supplemental Figure S1**

Phosphorylated CaMKIV (p-CaMKIV) is selectively expressed in Purkinje cells of the cerebellum. Cerebellar sections from three-week-old wildtype mouse were subjected to indirect immunofluorescence using antibodies to p-CaMKIV and Calbindin and the DNA dye DAPI and analyzed by fluorescence microscopy.

**
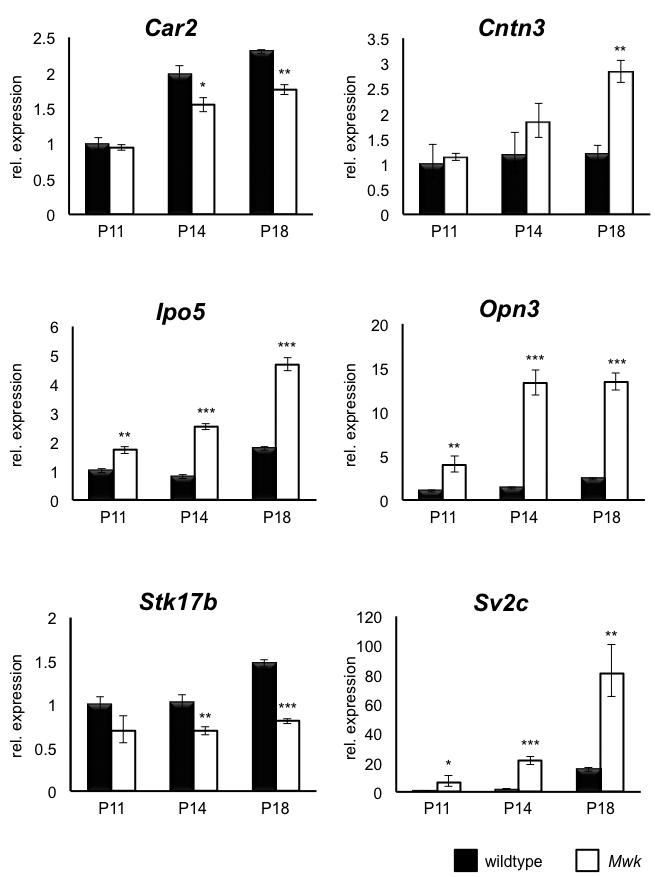
**

**Supplemental Figure S2**

Validation of microarray expression changes by quantitative real-time PCR (qRT-PCR). Timecourse of gene expression changes for selected candidate genes in Purkinje cells of *Mwk* mice compared to wildtype littermates at postnatal days P11, P14 and P18. qRT-PCR analysis was performed on four sets of LCM-isolated RNA for each genotype. Relative gene expression is shown; wildtype expression at P11 was set to 1.


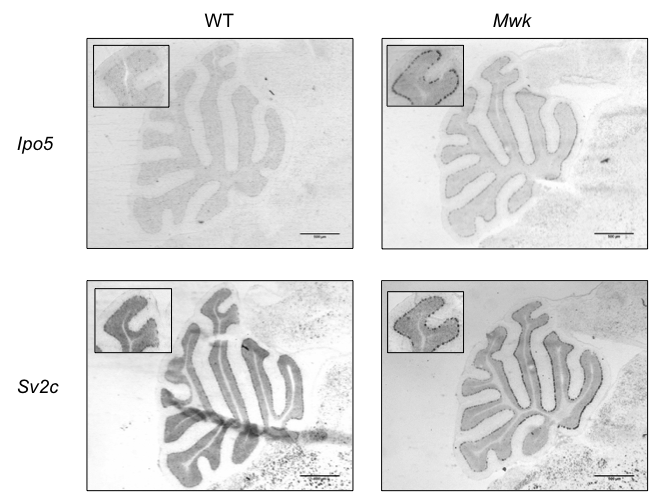


**Supplemental Figure S3**

Validation of microarray expression changes in sections of *Mwk* cerebellum at P18 compared to wildtype littermates by *in situ* hybridization. Two animals of each genotype were analyzed. Representative images are shown. Scale bar = 500 μm.


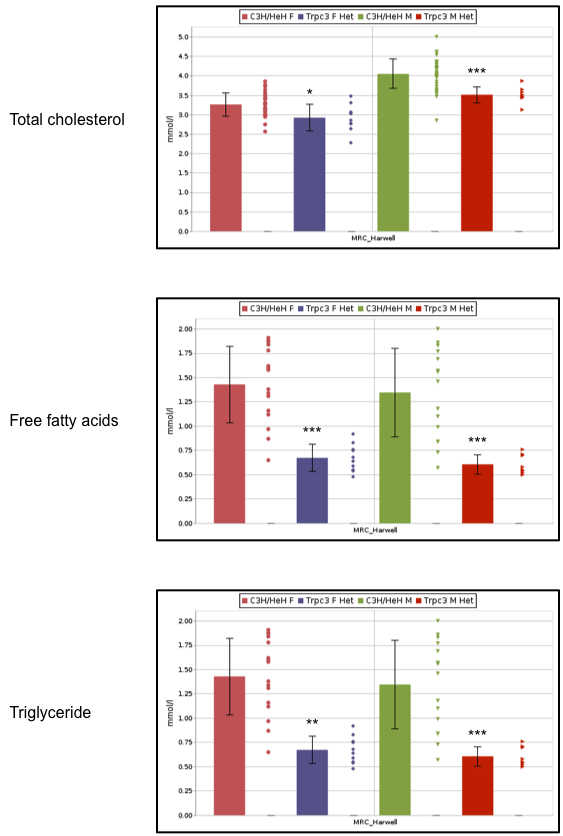


**Supplemental Figure S4**

Significant changes in serum levels of total cholesterol, free fatty acids and triglyceride between *Mwk* mice (Trpc3 Het) and wildtype controls (C3H/HeH). Mouse cohorts are split according to gender (F, M). Data are available from the Europhenome Mouse Phenotyping Resource (http://www.europhenome.org) (2).

**Supplemental Table S1**

List of 634 genes in *Mwk* Purkinje cells of 18-day-old mice that are significantly differentially expressed compared to wildtype littermates (*p*≤0.05).

| **Gene Symbol** | **qRT-PCR**  **P11** | **qRT-PCR**  **P14** | **qRT-PCR**  **P18** | **Microarray**  **P18** |
| --- | --- | --- | --- | --- |
| *Car2* | n.c. | -1.3±0.096 * | -1.3±0.056 ** | -2.8 |
| *Cntn3* | n.c. | n.c. | 2.4±0.11 ** | 2.2 |
| *Ipo5* | 1.7±0.103 ** | 3.1±0.057 *** | 2.6±0.045 *** | 3.2 |
| *Opn3* | 3.9±0.321 ** | 9.8±0.153 *** | 5.6±0.101 *** | 5.1 |
| *Stk17b* | n.c | -1.5±0.095 ** | -1.8±0.054 *** | -1.5 |
| *Sv2c* | 6.5±0.776 * | 12.1±0.198 *** | 5.3±0.311 ** | 4.4 |

**Supplemental Table S2**

Summary of significant gene expression changes in selected candidate genes. For each timepoint, Purkinje cell RNA from four different animals was isolated using LCM and subjected to qRT-PCR or microarray analysis, respectively. Standard errors are shown. *p<0.05; **p<0.01; ***p<0.001. n.c.: not changed.

**Supplemental Table S3**

Enrichment of differentially expressed genes in the Ingenuity functional category “Cell assembly and organization”.

**Supplemental Table S4**

Enrichment of differentially expressed genes in the Ingenuity functional category “Nervous system development and function”.

**Supplemental Table S5**

Top twenty diseases or functions annotations within the Ingenuity functional category “Neurological disease”.

**Supplemental Table S6**

Enrichment of differentially expressed genes in the Ingenuity functional category “Lipid metabolism”.

**Supplemental Table S7**

Top five canonical pathways enriched for differentially expressed genes.

**Supplemental Table S8**

Transcription factor (TF) enrichment among differentially expressed genes. Prediction analysis was performed using oPOSSum (3), TFactS (4), as well as available ENCODE Chip-Seq data from mouse cerebellum (http://encodeqt.stanford.edu) (5). TFs that were identified with more than one tool are highlighted in yellow.

**Supplemental References**

1. Becker, E.B.E., Oliver, P.L., Glitsch, M.D., Banks, G.T., Achilli, F., Hardy, A., Nolan, P.M., Fisher, E.M.C. and Davies, K.E. (2009) A point mutation in TRPC3 causes abnormal Purkinje cell development and cerebellar ataxia in moonwalker mice. *Proc. Natl. Acad. Sci. USA*, **106**, 6706–6711.

2. Morgan, H., Beck, T., Blake, A., Gates, H., Adams, N., Debouzy, G., Leblanc, S., Lengger, C., Maier, H., Melvin, D., *et al.* (2010) EuroPhenome: a repository for high-throughput mouse phenotyping data. *Nucleic Acids Res.*, **38**, D577–85.

3. Ho Sui, S.J., Mortimer, J.R., Arenillas, D.J., Brumm, J., Walsh,C.J., Kennedy, B.P. and Wasserman, W.W. (2005) oPOSSUM: identification of over-represented transcription factor binding sites in co-expressed genes. *Nucleic Acids Res.*, **33**, 3154–3164.

4. Essaghir, A., Toffalini, F., Knoops, L., Kallin, A., van Helden, J. and Demoulin, J.-B. (2010) Transcription factor regulation can be accurately predicted from the presence of target gene signatures in microarray gene expression data. *Nucleic Acids Res.*, **38**, e120–e120.

5. Auerbach, R.K., Chen, B. and Butte, A.J. (2013) Relating genes to function: identifying enriched transcription factors using the ENCODE ChIP-Seq significance tool. *Bioinformatics*, **29**, 1922–1924.
